# Cell-free DNA screening for sex chromosomal aneuploidies in 9985 pregnancies: Italian single experience

**DOI:** 10.1186/s13104-020-05009-1

**Published:** 2020-03-18

**Authors:** Katia Margiotti, Anthony Cesta, Claudio Dello Russo, Antonella Cima, Maria Antonietta Barone, Antonella Viola, Davide Sparacino, Alvaro Mesoraca, Claudio Giorlandino

**Affiliations:** 1 Human Genetics Lab, Altamedica Main Centre, Viale Liegi 45, 00198 Rome, Italy; 2 Department of Prenatal Diagnosis, Altamedica, Fetal-Maternal Medical Centre, Viale Liegi 45, 00198 Rome, Italy

**Keywords:** Sex chromosome aneuploidies (SCAs), NIPT, Next-generation sequencing, Cell-free fetal DNA (cffDNA)

## Abstract

**Objective:**

Non invasive prenatal testing (NIPT) using cell-free fetal DNA (cffDNA) has been widely accepted in recent years to detect common fetal autosomal chromosome aneuploidies and sex chromosome aneuploidies (SCAs). In this study, the clinical performance of our fetal DNA testing was investigated by analyzing the sex chromosome aneuploidy aberrations among 9985 pregnancies. The study was a retrospective analysis of collected NIPT data from the Ion S5 next-generation sequencing (NGS) platform obtained from Altamedica Medical Centre of Rome.

**Results:**

NIPT analysis of 9985 pregnancies revealed 31 cases with abnormal SCA results (0.31%). Among the 31 positive NIPT cases, 22 women agreed to undergo fetal karyotyping, whereas 9 refused further analyses. Of the 22 women verified by karyotyping analysis, 77.3% (17/22) were confirmed to be true positive SCAs, whereas 22.7% (5/22) were false positive. Among the true positive cases, 53.0% (9/17) were positive for monosomy X, 17.6% (3/17) were positive for 47, XXX aneuploidy, 23.5% (4/17) were positive for 47, XXY aneuploidy, and 5.9% (1/17) were positive for 47, XYY aneuploidy. In conclusion, the present results confirm that NIPT is a potential method for SCA screening, although this technology needs to be further investigated to improve the test performance.

## Introduction

The discovery of cell-free fetal DNA (cffDNA) in maternal plasma prompted the development of non invasive prenatal testing (NIPT), which introduced a new approach for screening common fetal aneuploidies into clinical practice, reducing unnecessary invasive procedures such as amniocentesis and chorionic villus sampling that may result in miscarriage or intrauterine infection [1]. NIPT is extensively based on targeted and whole-genome technologies using next-generation sequencing (NGS). NGS technologies have been developed and validated to detect increases in cell-free fetal DNA (cffDNA), which may depend on the presence of fetal chromosomal abnormalities [2–4]. The most common aneuploidies that occur at birth are trisomies 21, 18, and 13 and those involving the X and Y chromosomes. Chromosome abnormalities related to chromosome X or Y, also known as sex chromosome aneuploidies (SCAs), occur relatively frequently, with an overall rate of approximately 1/400 births, and aberrant chromosome count can cause male or female sexual organ dysplasia. SCAs have been associated with a series of diseases, such as Turner syndrome (monosomy X, 1/2000 in baby), Klinefelter syndrome (47, XXY, 1/500 in male), trisomy X (47, XXX, 1/2000 in female) and Jacob syndrome (47, XYY, 1/1000 in male). Approximately 99% of monosomy X conceptions spontaneously abort during pregnancy, while most other SCAs exhibit no clear clinical symptoms or abnormalities by ultrasound scan [5]. In addition, traditional procedures to detect fetal genetic chromosome aberrations require invasive procedures, especially chorionic villus sampling or amniocentesis, carrying a risk of fetal loss [6]. Therefore, an efficient and less invasive method for detecting SCAs is urgently needed in clinical practice. Most of the laboratories that offer NIPT include SCA testing as part of this test. According to the scientific literature, the detection accuracy of SCAs by NIPT assay varies among different research groups [7–9]. For example, Deng C. et al. [7] found that the PPV of NIPT was 18.39% for monosomy X, 44.4% for trisomy X, 39.29% for 47, XXY, and 75% for 47, XYY, while Zheng et al. [10] reported a PPV of 44.4% for monosomy X, 58.3% for trisomy X, 100% for 47, XXY, and 50% for 47, XYY. Although high sensitivity and specificity for the determination of fetal sex chromosome aneuploidies are presented by commercial laboratories, no clear and consistent data on SCA detection in pregnancies have been reported. Hence, in this study, we investigated the application of NIPT in SCA screening among pregnant women admitted to Altamedica Medical Centre (Rome, Italy) between January 2018 and January 2020. The overall aim of this study was to evaluate the clinical performance of our fetal DNA NIPT-NGS-based methodology in detecting sex chromosome aneuploidies in a cohort of 9985 samples.

## Main text

### Methods

#### Subjects

The study included a retrospective investigation of 9985 pregnant women who were admitted at Altamedica Medical Centre (Rome, Italy) and underwent a fetal cfDNA screening test between January 2018 and January 2020. The present study was approved by the local Ethics Committee of Artemisia SPA, and all participating women provided written informed consent.

#### Sample collection and NIPT analysis

In total, 9985 patients were tested using the Ion S5 NGS (ThermoFisher Scientific, Waltham, MA, USA) platform. The features of the patients and their indications for NIPT are summarized in Table 1. Protocol: Blood samples were collected in Streck Cell-Free DNA BCT tubes (Streck, City, State, USA), and samples were centrifuged on the same day. Approximately 3–4 ml plasma was isolated and stored at − 80 °C until cffDNA extraction. The cfDNA was extracted by using the Qiasymphony DSP Virus or Qiasymphony Circulating DNA Kit (Qiagen, Valencia, CA, USA). The DNA concentration was determined using the Agilent Technologies 4200 TapStation System (Agilent Technologies, Santa Clara, CA, USA) and the High Sensitivity D1000 ScreenTape System (Agilent Technologies, Santa Clara, CA, USA). The acceptable cffDNA range to process the sample was between 80 and 120 pg/µl. DNA libraries were prepared using the Ion AmpliSeq Kit according to the manufacturer’s instructions (ThermoFisher Scientific, Waltham, MA). The resulting libraries were sequenced using the Ion S5 system (Thermo Fisher Scientific, Waltham, MA). The results of the tests were provided to patients by post-test counseling; invasive diagnosis was offered for positive reports. Karyotyping by amniocentesis was selected as a method to confirm the NIPT results.

**Table 1:** Patient characteristics of 9985 pregnancies undergoing the NIPT test

Clinical characteristics	Mean value (range)
Maternal age	32.5 (20–50)
Body mass index, kg/m^2^	24.3 (14.5–43.5)
Gestational age, wk	16.3 (10–26)

Indications for NIPT	Ratios %
Maternal serum screening result	26.3
Advanced maternal age ≥ 35	41.2
Others (family history, IVF pregnancy, decision of perinatology council, etc.)	32.5

*wk* week, *IVF* in vitro fertilization, *NIPT* non invasive prenatal test

#### Data analysis and statistical analysis

Sequencing data were analyzed using a proprietary algorithm. Briefly, the detection of fetal aneuploidies is based on a combination of high-quality alignments against the human genome and read counts to identify chromosomal gains. A first normalization is performed based on GC percentage to calculate a high-quality z-score. The presence of a fetal aneuploidy for all 24 chromosomes is assessed by the value of the z-score calculations. The lack of results for a sample was attributed to an insufficient (< 4%) fraction of cffDNA or failure to pass the quality control parameters. Based on these results, chromosomal aneuploidy is determined in the fetus. The positive predictive value (PPV) was calculated with the Vassarstats online calculator (http://www.vassarstats.net). The required sample size was calculated using the GPower software, version 3.1.9.6 (Universitat Dusserldorf, Dusseldorf, Germany). An ideal sample size of at least 9200 was calculated (statistical power = 99%, α = 0.05, sensitivity/specificity = 90%) considering a prevalence of 0.25% of SCA in the population [11].

### Results and discussion

The objective of this study was to evaluate the performance of our fetal DNA test in detecting SCAs in a cohort of 9985 mixed-risk samples admitted to our laboratory between January 2018 and January 2020. The median gestational age in this testing cohort was 16.3 weeks. The median maternal age was 32.5 years. The median body mass index was 24.3 kg/m^2^ (Table 1).

The median fetal fraction of reported samples was 7.3%. The median time for reporting was 5 business days. A repeat sample was reported in 1.5% of the sample cohort, mainly due to insufficient fetal fraction (below a prespecified threshold of 4%), low reads, and discordant sex. In this cohort, the overall frequency for the SCA-positive NIPT test was 0.31% (Table 2). To assess the accuracy of positive NIPT results, follow-up based on confirmatory testing using amniocentesis for abnormal samples is recommended. Follow-up confirmation through fetal karyotyping was available for 22 NIPT-positive patients (13 for monosomy X; 3 for trisomy X; 5 for 47, XXY; and 1 for 47, XXY) (Table 2). Data on pregnancy outcome were missing for 9 NIPT-positive cases (6 for monosomy X; 1 for trisomy X; 1 for 47, XXY; and 1 for 47, XXY) because the women declined follow-up called “Unconfirmed” in Table 2. Based on the confirmatory follow-up, the PPV indicating the probability that fetuses with a positive test truly have the genetic disorder was available for 13 samples with monosomy X (Turner syndrome), 3 with trisomy X, 5 with 47, XXY (Klinefelter syndrome), and 1 with 47, XYY (Jacob syndrome) (Table 2). In particular, among the 13 cases with monosomy X, 9 were found to be true positive. Among the 4 cases with trisomy X, 3 cases were found to be true positive. Of the 6 cases of the 47, XXY karyotype, 4 cases were true positive. Finally, NIPT indicated 47, XYY in 2 cases, and karyotyping analysis confirmed 1 of the 2 cases as true positive. The estimated PPV for monosomy X was 69.2%, that for trisomy X was 100%, that for 47, XXY was 80%, and that for 47, XYY was 100%. The overall PPV of NIPT in the present study for fetal SCAs was 77.3% (Table 2). The detection accuracy of SCAs using NIPT varies greatly among different groups. For instance, Petersen et al. [12] found a PPV of 26% for monosomy X, 50% for trisomy X, and 86% for 47, XXY, while Zhang et al. [8] found a PPV of 29.41% for monosomy X, 100% for trisomy X, 77.78% for 47, XXY, and 100% for 47, XYY. Consistent with previous reports, monosomy X had the lowest PPV compared with the other SCAs in the present study [7, 9, 10], while the PPVs for monosomy X, trisomy X, 47, XXY, and 47, XYY aneuploidies were similar or even more accurate than that reported previously [10, 13–15].

**Table 2:** Efficiency of fetal DNA clinical testing for SCAs

SCA type	NIPT positive	Karyotype validated	True positive	False positive	PPV performance % (CI_95_)	Unconfirmed
Total SCAs	31	22	17	5	77.3 (54.2–91.3)	9
Monosomy X	19	13	9	4	69.2 (38.9–89.6)	6
Trisomy X	4	3	3	0	100 (31–100)	1
47, XXY	6	5	4	1	80 (29.9–98.9)	1
47, XYY	2	1	1	0	100 (5.5–100)	1

*PPV* positive predictive value

Cell-free DNA screening for aneuploidies by NGS-based methodologies has been widely used for SCAs in recent years, but clinical studies on its efficacy in Italy by single centers are lacking. Whereas the fetal DNA test is a NIPT test with high performance able to detect common autosomal trisomies, such as trisomy 13, 18, and 21, its validity with respect to sex chromosome aneuploidy detection is still controversial.

In this prospective study, we collected 9985 clinical samples and evaluated the NIPT strength by analyzing the cytogenetics outcome. Our test results were SCA positive in 31 samples. Of these 31 NIPT-positive cases, 22 were further evaluated by fetal karyotyping, and 17 samples were confirmed to have SCAs, showing an overall PPV of 77.3%. Our study represents a clinical experience of the NIPT test in Italy for fetal SCA detection. In our hands, the performance of NIPT in detecting sex chromosome aberrations was comparable to that of recently published clinical studies and, in some cases, was more accurate [9, 10]. This study has a number of limitations. First, due to the low incidence of SCAs in the general population (1/400 newborns) more pregnancies must be evaluated to better investigate and define the accuracy of this test for the detection of SCA syndromes. Secondly, since newborn with SCA syndrome can appear phenotypically normal, sensitivity, specificity, and negative predictive value could not be calculated, and so caution needs to be expressed in these type of studies, unless all neonates undergoes to karyotyping analysis.

### Conclusion

In the present study, we demonstrated that NIPT can be used for SCA detection by coupling the analysis of cell-free fetal DNA with next-generation sequencing methodology, although larger population studies are needed to better determine the accuracy of the test, particulary for monosomy X (45,X) in order to improve its performance. Therefore, NIPT in SCAs detection needs to be further investigated and validated.

## Limitations

Some limitations refer to the sample size and statistical performances. Out of 9985 cases only 31 cases had SCAs, this number need to be incremented to further investigate and validate SCA detection by NIPT methodology. Moreover, since it is not possible to perform the gold standard method for all patients (karyotype analysis), we cannot focus on the performance of the test in terms of sensitivity/specificity.

## Data Availability

Not applicable.

## Abbreviations

NIPT: Non invasive prenatal testing
cffDNA: Cell-free fetal DNA
SCAs: Sex chromosome aneuploidies
NGS: Next-generation sequencing
PPV: Positive predictive value
